# A Hospital as a Cinema Theatre

**Published:** 1912-02-24

**Authors:** 


*To the Editor of* The Hospital.

### A HOSPITAL AS A CINEMA THEATRE.

*To the Editor of* The Hospital.

Sir,—In your issue of February 17, a "District Surveyor" states that the article on the above subject, which appeared in The Hospital of February 10, is "rather misleading so far as London is concerned." He refers to the London Building Act, by s. 25 (5) of which (according to him) a hospital is defined as a public building. As a matter of fact the sub-section in question does not define a hospital. It merely states that the expression "public building" means (among a host of other things) "a building used or constructed or adapted to be used as . . . a hospital." Inasmuch as the phrase "public building" does not occur in the Cinematograph Act, 1909, it is difficult to see what bearing its definition has upon the discussion. Your correspondent also says (if one rightly understands his meaning) that the phrase "dwelling house," which is also explained in the defining section of the London Building Act, does not include a hospital. That, no doubt, is so; but the definitions set out in that Act explain the words as used in that particular statute. It is a private Act, applicable to London as a whole, and its definitions do not limit or elucidate the meaning of the same words as used in any Act of general application.

I am, Sir, etc.,

February 20. "Lex."

